# Health-Related Quality of Life of Omani Adult Patients with Sickle Cell Disease at the Sultan Qaboos University Hospital

**DOI:** 10.18295/squmj.10.2023.065

**Published:** 2024-08-29

**Authors:** Sara Al-Hinai, Asma Al-Rashdi, Hana Al-Sumri

**Affiliations:** 1 College of Medicine and Health Sciences, Sultan Qaboos University, Muscat, Oman.; 2 Department of Family Medicine and Public Health, Sultan Qaboos University, Muscat, Oman.

**Keywords:** Sickle Cell Disease, Health-Related Quality of Life, Adults, Oman

## Abstract

**Objectives:**

This study aimed to determine the health-related quality of life (HRQoL) of adult Omani patients with sickle cell disease (SCD). The quality of life of these patients in Oman is unknown.

**Methods:**

This cross-sectional study was conducted at the Sultan Qaboos University Hospital, Muscat, Oman, from June to October 2022 and included patients with SCD. A validated Arabic version of the 36-Item Short-Form Health Survey (SF-36) was used to assess HRQoL in 8 domains.

**Results:**

A total of 235 patients with SCD were included in this study, the majority of whom were female (74.9%) and between 18 and 35 years of age (64.6%). The lowest HRQoL was reported for the domain of role limitations due to physical health (median score = 25.0, interquartile range [IQR] = 35.0), while the highest was seen for role limitations due to emotional problems (median score = 66.7, IQR = 100.0). Frequent SCD-related emergency department visits/hospital admissions and the adverse effect of SCD on patients’ social lives had a significant negative impact on SF-36 scores for all 8 HRQoL domains (*P* ≤0.05). Additionally, SCD’s impact on academic performance and a history of having been bullied due to SCD had a significant negative impact on SF-36 scores for 7 domains (*P* ≤0.05).

**Conclusion:**

Omani adult patients with SCD reported relatively poor HRQoL in several domains, with various factors found to be significantly associated with this. Healthcare providers and policy makers should consider offering additional clinical, educational and financial support to these patients to enhance their HRQoL.

**Advances in Knowledge**

- *Study findings indicate a poor quality of life (QoL) among adult patients with sickle cell disease (SCD) in Oman in several domains of the 36-Item Short-Form Health Survey (SF-36) survey*.
- *Frequent SCD-related emergency department admissions, the adverse effect of SCD on patients’ social lives and academic performance and a history of having been bullied due to SCD had a significantly negative impact on their SF-36 scores in several domains*.
- *The QoL of Omani patients with SCD was previously unknown but has been measured in this study using a validated Arabic version of the widely used SF-36 QoL survey*.

**Application to Patient Care**

- *Healthcare providers should consider early screening for SCD-related complications to reduce the need for frequent hospitalisation*.
- *Policy makers should consider introducing protective and supportive legislation to reduce the social stigma surrounding this disease; this would improve the social life and academic performance of these patients and encourage acceptance of this patient group within the workforce and community*.

Haemoglobinopathies represent a significant public health issue worldwide; they are a group of inherited blood disorders that occur due to abnormalities in the synthesis and structure of haemoglobin molecules, which result in a reduction in the oxygen-carrying capacity of red blood cells.^1^^,^^2^ Sickle cell disease (SCD) is a very prevalent monogenic haemoglobinopathy occurring due to the substitution mutation of thiamine for adenine in the beta-chain gene, leading to the creation of haemoglobin S, a variant of the normal hemoglobin.^3^^,^^4^ This mutation causes haemoglobin polymerisation, resulting in irregular, crescent-shaped, sticky, rigid and fragile red blood cells.^4^ Patients experience a wide range of complications, including chronic pain, fatigue, cardiovascular events, leg ulcers and recurrent, unpredictable episodes of microcirculation obstruction resulting in multi-organ ischaemic damage and excruciating sickle cell-related pain, also referred to as vaso-occlusive crises.^5^ Other possible complications include stroke, pulmonary hypertension, acute chest syndrome, gallstones, priapism and pregnancy complications.^5^^,^^6^

Due to the chronic nature of SCD and the severity of SCD-related complications, affected individuals experience considerable disruption of their normal day-to-day lives and require frequent medical attention in the form of emergency department (ED) visits or hospitalisation.^7^^,^^8^ This burden is often compounded by a lack of awareness of the disease complications and an underestimation of patients’ needs by medical professionals, sometimes resulting in an over-reliance on ED care.^9^ Such factors contribute to poor health-related quality of life (HRQoL), a concept defined as the “patient’s appraisal of how his/her well being and level of functioning, compared to the perceived ideal, are affected by individual health”.^10^ Research shows that adult patients with SCD have poorer HRQoL compared to the general population as well as to individuals with other chronic diseases.^11^–^13^ Additionally, researchers have observed that SCD adversely impacts various aspects of patients’ lives, causing sleep disturbances and affecting their ability to keep a job, perform well academically and partake in normal social, recreational and daily activities, thereby affecting their marital, financial and employment prospects.^8^^,^^13^

A study of 629 Saudi adult patients with SCD reported that disease-related complications such as fever, skin redness or swelling and history of blood transfusion tend to adversely impact the HRQoL and 36-Item Short-Form Health Survey (SF-36) scores of SCD patients.^12^ In Oman, haemoglobinopathies are relatively common, with prevalence rates estimated to be 5.8% and 2.2% for the sickle-cell and beta-thalassaemia traits, respectively, and showed no significant gender differences.^14^ A recent study to assess health-related stigma, social support, self-care and self-efficacy among Omani adults with SCD found that most of them experienced a low level of stigma due to the social and psychological support provided by their families and their strong Islamic faith, although unmarried participants reported greater stigma in contrast to married participants.^15^ Nonetheless, there remains limited data on the HRQoL of Omani adults with haemoglobinopathies. Such research is crucial to reduce the burden on health resources and create policies to enhance the lives of affected patients and aid healthcare professionals in counselling and managing this chronic disease. Therefore, this study aimed to assess the HRQoL of Omani adults with SCD, using the Medical Outcomes SF-36 and determine the associations between patients’ measured HRQoL and selected demographic, social and clinical characteristics.^12^

## Methods

This cross-sectional study was conducted from June to October 2022 at the Sultan Qaboos University Hospital (SQUH), a tertiary care centre in Muscat, Oman. The target population of the study was adult patients of both genders and of Omani nationality aged 18 years or older who had been diagnosed with SCD and who were being followed-up at SQUH over the course of the study period. Patients diagnosed with other haemoglobinopathies, patients with SCD who were <18 years old, those who could not write or read, patients admitted due to vaso-occlusive crisis or surgery and those who were under the influence of sedative painkillers were excluded from the study.

Based on an estimated total number of patients with SCD being followed-up at SQUH (N = 1,000 registered patients in 2022), the necessary sample size for this study was calculated at 215, considering a confidence interval of 95% and a permissible rate of error of 5%. The mean and standard deviation of the outcome measure was taken from a previous study conducted in a neighbouring country.^13^

The SF-36 is a standardised, validated questionnaire used to assess HRQoL in 8 domains with a total of 36 items, including the respondent’s perception of their physical function (10 items), physical role health (4 items), emotional role functions (3 items), emotional well-being (5 items), social function (2 items), bodily pain (2 items), energy/fatigue (5 items) and general health perceptions (5 items).^16^

Data obtained from SF-36 were scored based on the scoring system reported by RAND Health. Each item in the SF-36 questionnaire is represented as a single variable and scaled from 0 to 100, with a higher score indicative of a better HRQoL. The original SF-36 has been shown to be a reliable measure of HRQoL, with Cronbach’s alpha values ranging from 0.78–0.93.^17^–^19^ The internal consistency and reliability of the SF-36 has been investigated in a previous pilot study of 80 patients with SCD in Saudi Arabia. A high internal consistency (Cronbach’s alpha>0.6) has been reported for physical function (Cronbach’s alpha=0.81), physical role health (Cronbach’s alpha=0.84), emotional role functions (Cronbach’s alpha=0.86), vitality (Cronbach’s alpha=0.79), emotional well-being (Cronbach’s alpha=0.67), social function (Cronbach’s alpha=0.67), bodily pain (Cronbach’s alpha=0.84) and general health (Cronbach’s alpha=0.60).^12^ For the purpose of the present study, this already validated Arabic-language version of the SF-36 was used.^12^

Another 2-part questionnaire was developed to collect data regarding the participants’ demographic, social and clinical characteristics. The first section assessed the demographic background of the participants (gender, age, place of residence, education level and employment and marital status). The second section collected information regarding the participants’ clinical characteristics (frequency of hospital admissions, blood transfusion and disease-related complications) and their perceived social impact of the disease (impact on marriage, social and academic life). This included a recent history of ED visits or hospital admissions over the past 3 months, the presence of medical complications and existence of family support among other items.

A link to the online survey was distributed electronically to all eligible patients with SCD, who were identified using the hospitals’ registry. The questionnaire’s QR code was shared with participants, and it was self-administered. All Omani adult patients diagnosed with SCD and being followed-up in the haematology outpatient department during the study period were invited to participate in the study. A study information sheet was provided for participants in their preferred language, prior to questionnaire distribution. To avoid duplicated data, a researcher ensured that each participant only filled out the questionnaire once, using each participant’s date of birth as an identifier.

Descriptive data are presented as percentages for categorical variables. Continuous variables are presented as either mean and standard deviation or as medians and interquartile ranges, depending on the normality of the data distribution which was determined using a one-sample Kolmogorov-Smirnov test. Scores for each of the 8 HRQoL domains were calculated using the SF-36 scoring index and presented as either means or medians, depending on the normality of the data distribution. Bivariate associations between mean/median SF-36 scores for each of the HRQoL domains and various demographic, clinical and social impact factors were calculated using either parametric or non-parametric statistical tests. Analyses were conducted using the Statistical Package for the Social Sciences (SPSS) (IBM Corp., Armonk, NY). A *P* value of ≤0.05 was considered statistically significant.

All participants provided consent to take part in this study; this was done via a consent statement which was attached to the questionnaire link and had to be ticked by the participants who agreed to take part in the study. Ethical approval for this study was obtained from the Medical Research and Ethics Committee of the College of Medicine and Health Sciences, Sultan Qaboos University (REF. NO. SQU-EC/153/2022).

## Results

A total of 235 Omani adult patients with SCD participated in the study, of which the majority were female (74.9%) and aged 18–25 (26.5%) or 25–35 (38.1%) years. Most of the patients resided in either Muscat (39.7%) or Ad Dhakhiliyah governorate (23.7%). The majority were married (53.4%), with only 2 patients being divorced (0.8%) and 1 (0.4%) being widowed; the remaining 107 patients (45.3%) were single. Regarding education, most patients had a college diploma, bachelor’s degree or postgraduate qualification (69.1%). Over one-third of the sample were unemployed (14.6%) or currently seeking work (24.9%).

**Figure 1:**
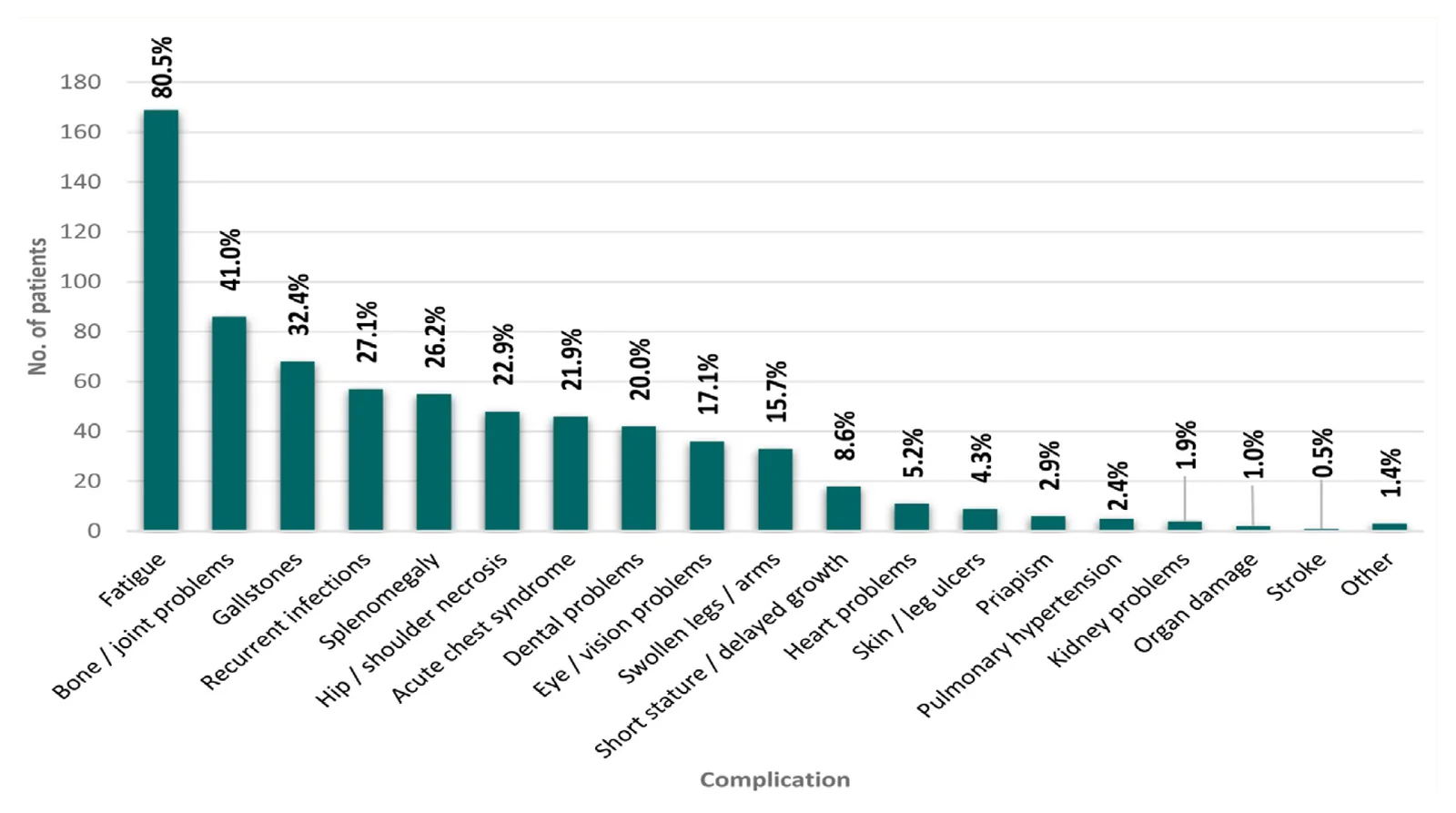
Figure 1: Frequency of sickle cell disease-related complications among Omani adult patients with sickle cell disease (N = 235)^*^ ^*^Percentages do not add up to 100% as patients may have had more than 1 complication.

Regarding clinical characteristics, most of the patients (61.3%) had a history of recent ED visits or hospital admissions due to SCD, with 35.8%, 13.2%, 3.3% and 9.0% reporting 1–2, 3–4, 5–6 and ≥7 visits/admissions in the preceding 3 months, respectively. Additionally, 46.5% of the patients had received at least 1 exchange blood transfusion, while 16.2% had undergone a splenectomy [Table 1]. Fatigue was the most frequently reported SCD-related disease complication (80.5%), followed by bone/joint problems (41.0%), gallstones (32.4%), recurrent infections (27.1%), splenomegaly (26.2%), hip/shoulder necrosis (22.9%) and acute chest syndrome (21.9%) [Figure 1]. Additionally, the majority of the married female patients (68.7%) reported having difficulties conceiving or complications during pregnancy. A proportion of patients also reported mental health difficulties, with anxiety being most common (23.0%), followed by sleep disturbances (18.7%), depression (11.9%) and social isolation (6.8%).

Regarding the social impact of SCD, 44.6% of the single respondents reported that the disease had played some role in delaying marriage; however, only 24.8% of the married participants claimed that SCD had caused them marital issues. Just over one-third (36.8%) of participants with children stated that SCD had negatively affected either their relationship with their children or their ability to care for them. Most of the patients reported that SCD had negatively affected their academic performance (64.9%) and limited their social life (64.2%). Additionally, 34.5% of the patients claimed that they had either been bullied or had their abilities belittled due to SCD [Table 2].

Of the 8 HRQoL domains assessed by the SF-36 tool, the highest median score (66.7) was reported for the domain of role limitations due to emotional problems, indicating that the patients experienced better quality of life in this domain. The next highest scoring HRQoL domain was social functioning (median SF-36 score: 62.5), followed by physical functioning and emotional well-being (median SF-36 score: 60.0 each). In turn, the domain with the lowest median score, denoting the poorest HRQoL, was that of role limitations due to physical health (median SF-36 score: 25.0), followed by the energy/fatigue domain (median SF-36 score: 50.0). Patients reported relatively neutral/better quality of life for the remaining 2 HRQoL domains—bodily pain and general health (median SF-36 score: 55.0 each) [Table 3].

Overall, there was no significant association between any of the selected sociodemographic characteristics and the 8 HRQoL domains, except for employment status, for which a significant association was observed in the social functioning domain (*P* = 0.009) [Table 4]. However significant associations were observed between the number of disease complications experienced by the patient and 6 HRQoL domains, with patients with a greater number of complications reporting poorer HRQoL in the domains of physical functioning (*P* = 0.005), bodily pain (*P* = 0.003), general health (*P* = 0.026), social functioning (*P* = 0.006) and role limitations due to physical health (*P* = 0.014) and emotional problems (*P* = 0.018).

A history of pregnancy difficulties was significantly associated with lower scores in the HRQoL domains of pain (*P* <0.001), general health (*P* = 0.007) and role limitations due to physical health (*P* = 0.032). Patients with a history of exchange blood transfusions scored significantly lower in the domains of physical functioning (*P* = 0.020), energy/fatigue (*P* = 0.033), pain (*P* <0.001), general health (*P* = 0.043), social functioning (*P* = 0.046) and role limitations due to physical health (*P* = 0.019), while those with a history of blood transfusions reported experiencing worse bodily pain (*P* = 0.007). Patients’ number of recent ED visits/hospital admissions was a crucial factor observed to be associated with significantly poorer HRQoL scores across all 8 domains (*P* ≤0.05 each).

Patients who reported being compliant with their prescribed medications (*P* = 0.021) and those who received family support (*P* = 0.006) had lower scores in the pain HRQoL domain. Patients who exercised more frequently reported better HRQoL in the physical functioning domain (*P* = 0.031). Regarding the perceived social impact of the disease, patients who reported poor academic performance and those who experienced bullying due to SCD both had significantly poorer scores in all HRQoL domains apart from the physical functioning domain (*P* <0.05 each). Importantly, the perception that SCD had resulted in limitations to the patients’ social life was significantly associated with poorer scores in all 8 HRQoL domains (*P* ≤0.05) [Table 5].

## Discussion

Research focusing on HRQoL is essential to ensure the provision of personalised and effective patient care, reduce the onset of complications, advocate for policy changes and ultimately help to improve the lives of individuals living with chronic conditions, including SCD.^20^^,^^21^ As such, this study aimed to evaluate the HRQoL of Omani adult patients with SCD and determine its associations with various sociodemographic and clinical characteristics.

Of the 8 HRQoL domains assessed, the present study found that Omani adults with SCD reported having the poorest quality of life in the domain of role limitations due to physical health (median SF-36 score: 25.0). A similar study reported comparable findings, in which this domain received the lowest score (mean SF-36 score: 35.9) among adult patients with SCD being followed-up in 2 hospitals in 2 different regions of Saudi Arabia.^12^ Omani patients with SCD in the current study reported having the highest quality of life in the domain of role limitations due to emotional problems (median SF-36 score: 66.7), while the Saudi Arabian study reported the best HRQoL for the domain of social functioning (mean SF-36 score: 60.1) which was the second highest domain in the present study (median SF-36 score: 62.5).^12^ In general, however, Omani patients in the current study reported having poor HRQoL in multiple domains, considering a cut-off score of 50 to represent a distinction between poor and good HRQoL.^16^^,^^22^

As in most Arab countries, the practice of early and universal marriage is still highly prevalent in Oman; essentially, this means that majority of individuals aged >20 years are expected to be married.^23^ Thus, the fact that almost half of the sample (45.3%) in the present study were single represents a considerable deviation from sociocultural norms. The study from Saudi Arabia similarly reported that 55.6% of the adult patients with SCD were unmarried, although another study indicated a much lower rate of single participants (27.5%) among a cohort of patients with SCD attending multiple primary care centres in Bahrain.^12^^,^^24^ Nonetheless, despite the high prevalence of unmarried patients, marital status was not found to be significantly associated with HRQoL in the current study. Similarly, no associations were determined between HRQoL and most of the other sociodemographic factors, such as gender and age; this is consistent with the findings of the aforementioned Saudi Arabian study.^12^ Previous research has indicated that age might be related to poorer quality of life among adolescents with SCD, particularly in domains related to social functioning, possibly due to higher rates of actual or perceived stigmatisation by their peers.^25^–^27^ In contrast, another study noted that among a cohort of young adults with SCD, increasing disease severity trajectories over time were associated with older age, a factor linked with higher risk of SCD complications and increased care requirements, likely resulting in poor HRQoL.^28^

Indeed, the frequent SCD complications observed in the current study include fatigue (80.5%), bone/joint problems (40.1%) and gallstones (32.4%), findings which are relatively similar to those of a previous study conducted in the USA, which reported frequent digestive and musculoskeletal complications among patients with SCD.^29^ As expected, a greater number of complications, more frequent ED visits or hospitalisations and a history of blood/exchange blood transfusions were factors found to significantly worsen HRQoL in the present study, all of which can be considered indications of disease severity. Additionally, the present study found that the majority (68.7%) of the married female patients with SCD complained of difficulties conceiving and/or pregnancy complications, with a significantly lower quality of life in several domains, including the general health, pain and role limitations due to physical function domains.

The present study identified a significant association between employment status and HRQoL scores in the domain of social functioning, with unemployed patients reporting significantly poorer scores compared to those who were either employed, students or job seekers. This finding aligns with that of previous research, indicating that unemployment is significantly linked to worse social functioning among patients with SCD.^28^^,^^29^ Employment is believed to be a crucial mediator of quality of life among patients with chronic diseases likely because it impacts multiple domains of life and provides individuals with a sense of purpose and value, fostering personal identity and self-esteem and promoting financial independence and material security.^30^ Despite frequent hospital admissions and complications, SCD did not appear to have affected the current study’s patients’ level of academic attainment, given that the majority of them (69.8%) had achieved a college diploma or higher degree. However, the high rate of unemployment or job-seeking (39.5%) indicates that securing a job remained a challenge due to their medical condition. Moreover, although no direct association was observed between HRQoL and education level, the patients reported a significant impact with regards to the perceived negative impact of the disease on their academic performance, including missing classes, being unable to participate in various school activities and performing poorly academically due to their medical condition.

Overall, patients who reported having social restrictions due to SCD also reported significantly poorer HRQoL across all 8 domains. This is likely due to an overlap or interaction between various HRQoL domains; for instance, a previous study reported a significant association between pain and social functioning.^29^ Patients in greater pain, indicating a more severe disease course, are likely to have lower physical functioning, vitality, general health and emotional well-being, all of which can impact their social lives in various ways. Al-Raqaishi *et al*. noted that patients with SCD often suffered the effects of health-related stigma.^15^ Similarly, just over one-third (34.5%) of patients in the present study reported having been bullied or belittled due to their condition, a factor which significantly affected their scores in various HRQoL domains. This finding is comparable to that of another study conducted in Nigeria, in which 26% of adults with SCD experienced teasing or bullying, with 66% perceiving negative societal attitudes towards the disease.^26^

To the best of the authors’ knowledge, this is the first study in Oman to measure the HRQoL of Omani adult patients with SCD. However, certain limitations must be acknowledged. First, the data collection tool was self-administered, and therefore, dishonest responses cannot be discounted. Additionally, the study was conducted in a single centre; thus, the results might not be representative of all Omani adult patients with SCD.

## Conclusion

The sample of Omani adult patients with SCD studied generally reported poor quality of life in multiple HRQoL domains, particularly in the role limitations due to physical health and energy/fatigue domains. Additionally, significant associations were observed between poorer HRQoL and various clinical and social characteristics, indicating that healthcare providers should pay more attention to patients with SCD during consultations and screen early for complications to enhance patients’ HRQoL. Moreover, policy makers should consider introducing protective legislation to reduce the social stigma surrounding this disease and encourage the acceptance of this patient group within the workforce and community.

## Figures and Tables

**Table 1:** Characteristics of Omani adult patients with sickle cell disease (N = 235)

Characteristic	n (%)*
**Gender (n = 235)**	
Male	59 (25.1)
Female	176 (74.9)
**Age in years (n = 235)**	
18–25	60 (26.5)
25–35	86 (38.1)
35–45	54 (23.9)
>45	26 (11.5)
**Place of residence (n = 226)**	
Muscat	92 (39.7)
Ad Dhakhiliyah	55 (23.7)
Al Batinah	39 (16.8)
Ash Sharqiyah	30 (12.9)
Ad Dhahirah	10 (4.3)
Al Buraimi	4 (1.7)
Dhofar	2 (0.9)
**Marital status (n = 235)**	
Single	107 (45.3)
Married	125 (53.4)
Divorced	2 (0.8)
Widowed	1 (0.4)
**Education level (n = 235)**	
Elementary	3 (1.3)
Middle school	7 (3.0)
High school	61 (26.0)
Diploma	45 (19.1)
Bachelor	103 (43.8)
Postgraduate	16 (6.8)
**Employment status (n = 233)**	
Student	41 (17.6)
Un-employed	106 (45.5)
Employed	86(36.9)
**Recent ED visits/hospital admissions due to SCD (n = 212)** †	
0	82 (38.7)
1–2	76 (35.8)
3–4	28 (13.2)
5–6	7 (3.3)
≥7	19 (9.0)
**History of exchange transfusions (n = 230)**	
Yes	107 (46.5)
No	123 (53.5)
**No. of exchange transfusions (n = 103)**	
1–2	44 (42.7)
3–4	21 (20.4)
≥5	38 (36.9)
**History of splenectomy (n =228)**	
Yes	37 (16.2)
No	191 (83.8)

ED = emergency department; SCD = sickle cell disease.

* Percentages were calculated out of the valid responses only due to missing data (<5%).

† Within the past 3 months.

**Table 2:** Frequency of responses to survey items relating to the social impact of sickle cell disease among Omani adult patients with sickle cell disease (N = 235)

Item	n (%)*
**If not yet married, was SCD a reason for delaying marriage? (n = 101)**	
Yes	45 (44.6)
No	56 (55.4)
**If married, has SCD caused any marital issues? (n = 109)**	
Yes	27 (24.8)
No	82 (75.2)
**If you have children, has SCD negatively affected your relationship or your ability to take care of them? (n = 87)**	
Yes	32 (36.8)
No	55 (63.2)
**Has SCD impacted your academic performance (e.g. absences/lower grades/failing/not participating in school activities)? (n = 225)**	
Yes	146 (64.9)
No	79 (35.1)
**Has SCD limited your social life (e.g. attending gatherings/parties with family and friends/travelling/participating in sports/clubs/other hobbies)? (n = 226)**	
Yes	145 (64.2)
No	81 (35.8)
**Have you been bullied/belittled by others due to SCD? (n = 226)**	
Yes	78 (34.5)
No	148 (65.5)

SCD = sickle cell disease.

* Percentages were calculated out of the valid responses only due to missing data (<5%).

**Table 3:** Health-related quality of life scores per domain among Omani adult patients with sickle cell disease (N = 235)*

HRQoL Domain	Score	
	Median†	IQR
Physical functioning	60.0	35.0
Role limitations due to physical health	25.0	35.0
Role limitations due to emotional problems	66.7	100.0
Energy/fatigue	50.0	25.0
Emotional wellbeing	60.0	27.0
Social functioning	62.5	38.0
Pain	55.0	43.0
General health	55.0	25.0

HRQoL = health-related quality of life; IQR = interquartile range.

* Using the 36-Item Short-Form Health Survey.

† Scores are presented as medians because the data did not follow a normal distribution according to a one-sample Kolmogorov-Smirnov test.

**Table 4:** Associations between selected characteristics and health-related quality of life domain scores among Omani adult patients with sickle cell disease (N = 235)*

Characteristic	HRQoL domain, Mean score ± SD or mean rank†							
	Physical functioning	Energy/fatigue	Pain	General health	Role limitations due to physical health	Role limitations due to emotional problems	Emotional well-being	Social functioning
**Gender**								
Male	114.41	100.97	103.71	95.11	100.11	112.59	105.90	101.16
Female	101.88	105.74	110.84	113.07	105.31	107.14	108.70	107.62
P value	0.194‡	0.615‡	0.462‡	0.064‡	0.574‡	0.557‡	0.774‡	0.496‡
**Age in years**								
18–25	106.67	105.10	57.98 ± 28.3	51.47 ± 16.9	106.41	106.47	101.91	110.76
25–35	98.36	96.55	57.56 ± 26.8	55.06 ± 16.4	101.38	106.41	104.81	104.12
35–45	99.59	99.04	51.01 ± 27.9	52.98 ± 18.2	96.91	91.76	100.17	86.71
>45	95.08	108.58	58.91 ± 27.1	55.87 ± 14.4	91.05	120.83	114.41	107.76
P value	0.828§	0.771§	0.464¶	0.168¶	0.697§	0.208§	0.804§	0.173#
**Place of residence**								
Muscat	58.41 ± 23.3	106.71	56.71 ± 29.7	52.47 ± 16.1	102.06	105.50	60.56 ± 20.4	99.51
Ash Sharqiyah	54.00 ± 27.2	112.65	58.10 ± 23.5	54.04 ± 19.1	104.42	107.68	57.69 ± 20.0	80.76
Dhofar	80.00	119.50	88.75 ± 15.9	67.50 ± 3.5	168.50	170.50	66.00 ± 19.8	183.00
Ad Dhahirah	56.88 ± 29.8	109.50	42.78 ± 27.2	51.25 ± 5.8	103.39	113.61	53.50 ± 14.8	122.78
Ad Dhakhiliyah	55.61 ± 23.0	96.72	49.29 ± 27.6	52.75 ± 18.5	99.89	108.91	63.00 ± 17.4	114.44
Al Buraimi	35.00 ± 7.1	90.50	56.25 ± 32.6	52.50 ± 15.5	87.63	62.25	61.00 ± 17.4	98.25
Al Batinah	62.64 ± 20.0	100.73	23.7	55.00 ± 16.8	106.27	106.97	60.34 ± 19.3	110.19
P value	0.597¶	0.927§	0.077¶	0.947¶	0.781§	0.546§	0.704¶	0.092#
**Marital status**								
Single	116.41	104.69	109.34	106.36	114.95	115.36	110.71	113.35
Married	97.11	105.97	110.19	112.49	96.12	104.15	105.72	101.25
Divorced	122.75	64.75	76.25	28.00	96.75	105.25	169.00	94.75
Widowed	12.50	-	-	-	125.00	89.00	-	-
P value§	0.052	0.629	0.749	0.142	0.140	0.571	0.327	0.336#
**Education level**								
Elementary	45.00 ± 42.4	56.00	45.00	57.50 ± 0.0	82.25	89.00	48.00 ± 5.7	11.00
Middle school	45.00 ± 23.8	84.60	51.43 ± 17.1	40.83 ± 39.2	114.50	102.71	57.14 ± 11.9	110.75
High school	56.70 ± 20.8	97.67	49.91 ± 21.8	51.45 ± 29.3	97.88	93.63	57.67 ± 20.0	92.26
Diploma	53.13 ± 20.8	108.06	59.01 ± 15.4	53.57 ± 28.5	91.54	105.75	58.93 ± 17.6	109.79
Bachelor	60.97 ± 25.5	108.07	59.45 ± 20.6	54.47 ± 25.7	111.12	116.73	62.23 ± 20.3	115.45
Postgraduate	62.67 ± 21.9	97.67	44.67 ± 19.7	53.75 ± 24.5	114.60	123.93	62.40 ± 12.7	89.50
P value	0.241¶	0.771§	0.167¶	0.306¶	0.431§	0.242§	0.329¶	0.109#
**Employment status**								
Student	118.71	102.01	124.43	116.65	126.61	121.73	63.89 ± 22.3	130.82
Employed	95.32	105.62	108.25	108.10	97.19	111.64	59.94 ± 18.9	93.36
Self-employed	101.25	96.22	73.56	111.67	104.06	108.56	54.67 ± 20.3	76.39
Unemployed	92.34	91.70	90.81	95.92	90.08	87.93	57.76 ± 16.6	91.37
Seeking a job	116.67	106.54	110.79	102.44	106.96	103.40	60.08 ± 18.4	116.25
Retired	104.79	117.67	115.38	126.63	88.59	120.50	63.00 ± 17.8	110.75
P value	0.222§	0.814§	0.148§	0.622§	0.092§	0.214§	0.796¶	0.009#

HRQoL = health-related quality of life; SD = standard deviation.

* Assessed using the 36-Item Short-Form Health Survey.

† SD values could not be calculated for variables that did not follow a normal distribution.

‡ Using the Mann-Whitney U test for non-normally distributed variables with 2 categories.

§ Using the Kruskal Willis test for non-normally distributed variables with ≥3 categories.

¶ Using the analysis of variance test for normally distributed variables with ≥3 categories.

**Table 5:** Associations between selected clinical and social variables and health-related quality of life domain scores among Omani adult patients with sickle cell disease (N = 235)*

Variable	HRQoL domain, Mean score ± SD or mean rank†							
	Physical functioning	Energy/fatigue	Pain	General health	Role limitations due to physical health	Role limitations due to emotional problems	Emotional well-being	Social functioning
**Complications (n)**								
1–3	102.26	50.92 ± 16.2	109.06	105.00	100.08	107.17	60.75 ± 18.3	107.18
4–6	97.35	47.73 ± 19.8	94.16	97.57	95.72	91.47	57.82 ± 20.4	89.94
≥7	57.53	40.94 ± 25.7	61.11	65.38	61.92	73.50	55.53 ± 15.8	66.31
*P* value	0.005§	0.097¶	0.003§	0.026§	0.014§	0.018§	0.417¶	0.006§
**History of pregnancy difficulties**								
Yes	48.86 ± 19.6	43.57	40.70	42.92	43.22	46.82	59.08 ± 18.9	44.40
No	59.46 ± 27.1	46.61	63.52	59.55	55.77	52.38	59.86 ± 19.9	53.35
*P* value	0.069\\	0.603‡	<0.001‡	0.007‡	0.032‡	0.341‡	0.855\\	0.138‡
**History of exchange transfusions**								
Yes	95.19	95.65	93.96	99.72	94.61	100.75	106.00	97.77
No	114.70	113.41	123.68	116.93	113.48	116.18	110.74	114.29
*P* value‡	0.020	0.033	<0.001	0.043	0.019	0.056	0.577	0.046
**History of blood transfusions**								
Yes	106.47	104.04	101.48	104.91	101.94	109.68	108.44	107.32
No	103.33	105.61	126.58	116.66	108.71	105.83	106.99	102.76
*P* value‡	0.728	0.864	0.007	0.201	0.438	0.659	0.875	0.616
**Recent ED visits/hospital admissions due to SCD** **								
0	66.62 ± 23.5	54.41 ± 18.1	146.27	59.37 ± 16.8	117.39	118.31	67.45 ± 17.3	129.82
1–2	56.39 ± 19.6	51.14 ± 17.6	92.65	52.81 ± 15.2	95.01	104.30	60.05 ± 19.2	93.55
3–4	51.20 ± 22.7	42.69 ± 19.9	61.95	45.71 ± 18.1	79.70	73.96	53.57 ± 20.7	63.98
5–6	48.33 ± 18.1	37.50 ± 17.5	61.33	36.67 ± 17.8	64.00	55.00	43.33 ± 18.8	83.00
≥7	46.39 ± 25.9	47.37 ±17.7	48.50	44.17 ± 12.4	73.83	90.69	54.12 ± 12.2	74.55
*P* value	<0.001¶	0.024¶	<0.001§	<0.001¶	0.001§	0.001§	<0.001¶	<0.001§
**Compliance with medications**								
Yes	56.11 ± 23.0	95.82	95.82	100.37	95.82	99.12	59.45 ± 19.6	58.37
No	59.44 ± 23.7	102.46	115.87	105.10	100.84	107.71	60.91 ± 18.6	101.71
*P* value	0.337\\	0.438‡	0.021‡	0.583‡	0.542‡	0.299‡	0.609\\	0.692‡
**Impact on academic performance**								
Yes	99.16	94.92	90.18	93.49	92.81	97.14	100.01	94.53
No	111.76	117.65	139.94	132.34	119.86	124.71	117.81	122.93
*P* value‡	0.148	0.009	<0.001	<0.001	0.001	0.001	0.045	0.001
**Impact on social life**								
Yes	96.39	96.38	91.17	93.38	91.57	96.66	100.28	92.96
No	119.81	117.34	139.41	133.14	124.57	127.59	119.37	127.92
*P* value‡	0.007	0.016	<0.001	<0.001	<0.001	<0.001	0.030	<0.001
**History of being bullied/belittled**								
Yes	100.60	88.45	92.86	88.02	86.78	87.95	85.57	90.41
No	106.61	111.94	116.28	118.01	112.56	118.49	118.89	113.51
*P* value‡	0.491	0.006	0.008	0.001	0.002	<0.001	<0.001	0.007
**Family support**								
Yes	104.70	102.23	100.93	102.73	100.80	106.88	108.24	103.99
No	98.66	100.94	130.44	119.17	104.23	102.45	94.52	101.48
*P* value‡	0.560	0.905	0.006	0.117	0.728	0.657	0.190	0.807
**Exercise frequency per week**								
0	95.03	99.73	102.84	51.59 ± 16.5	96.89	107.00	109.70	103.02
1	128.40	111.15	117.42	56.72 ± 16.7	117.83	122.81	101.16	110.20
2	112.94	103.59	108.70	55.28 ± 18.4	113.05	106.71	107.85	106.89
≥3	114.56	122.43	130.72	54.20 ± 15.2	109.30	100.72	107.48	114.90
*P* value	0.031§	0.360§	0.183§	0.465¶	0.209§	0.501§	0.930§	0.803§

HRQoL = health-related quality of life; SD = standard deviation; ED = emergency department; SCD = sickle cell disease.

* Using the 36-Item Short-Form Health Survey.

† SD values could not be calculated for variables that did not follow a normal distribution.

‡ Using the Mann-Whitney U test for non-normally distributed variables with two categories.

§ Using the Kruskal Willis test for non-normally distributed variables with ≥3 categories.

¶ Using the analysis of variance test for normally distributed variables with ≥3 categories.

\\ Using the independent sample t-test for normally distributed variables with 2 categories.

** Within the past 3 months.
